# XPS Study in BiFeO_3_ Surface Modified by Argon Etching

**DOI:** 10.3390/ma15124285

**Published:** 2022-06-17

**Authors:** Grecia Alejandra Gomez-Iriarte, Arbelio Pentón-Madrigal, Luiz Augusto Sousa de Oliveira, João Paulo Sinnecker

**Affiliations:** 1 Centro Brasileiro de Pesquisas Físicas, Rua Xavier Sigaud 150, Rio de Janeiro 22290-180, RJ, Brazil; 2 Facultad de Física, Universidad de La Habana, La Habana CP10400, Cuba; arbelio@fisica.uh.cu; 3 Núcleo Multidisciplinar de Pesquisas em Nanotecnologia, Universidade Federal do Rio de Janeiro, Rodovia Washington Luiz, km 104, 5, Duque de Caxias 25240-005, RJ, Brazil; laso@ufrj.br

**Keywords:** multiferroics, XPS, BiFeO_3_, low-pressure argon deposition, argon etching

## Abstract

This paper reports an XPS surface study of pure phase BiFeO${}_{3}$ thin film produced and later etched by pure argon ions. Analysis of high-resolution spectra from Fe 2p, Bi 4f and 5d, O 1s, and the valence band, exhibited mainly Fe${}^{3+}$ and Bi${}^{3+}$ components, but also reveal Fe${}^{2+}$. High-energy argon etching induces the growth of Fe${}^{\left(0\right)}$ and Bi${}^{\left(0\right)}$ and an increment of Fe${}^{2+}$, as expected. The BiFeO${}_{3}$ semiconductor character is preserved despite the oxygen loss, an interesting aspect for the study of the photovoltaic effect through oxygen vacancies in some ceramic films. The metal-oxygen bonds in O 1s spectra are related only to one binding energy contrary to the split from bismuth and iron reported in other works. All these data evidence that the low-pressure argon atmosphere is proved to be efficient to produce pure phase BiFeO${}_{3}$, even after argon etching.

## 1. Introduction

Multiferroics are multifunctional materials that combine two or more ferroic properties, whether electrical, magnetic or crystal deformation [1]. However, it is not usual to find these multiferroic materials in nature due to the intrinsic mechanisms that lead to ferroic properties; most ferromagnetic materials exhibit partial filled 3d levels, whereas ferroelectric materials show empty 3d levels (mainly in perovskites) [2]. Even though some multiferroics present mechanisms for getting ferroic properties combined [2,3], only a few show these properties at room temperature in the pure phase, the desired aspect for technological applications. Examples are the piezoelectric material lead zirconate titanate (PbZr${}_{x}$Ti${}_{1-x}$O${}_{3}$ or PZT) [4] and, bismuth ferrite (BiFeO${}_{3}$ or BFO). BiFeO${}_{3}$ is a multiferroic material that exhibits at room temperature ferroelectric and antiferromagnetic properties, with T${}_{C}$∼810–830 ${}^{\circ }$C and T${}_{N}$∼370 ${}^{\circ }$C respectively [5]. Magnetism comes from iron metal transition and electrical polarization comes from displacement of lone pair Bi 6s [2,3].

Despite these appealing properties, the pure phase BFO is difficult to synthesize thanks to its high sensitivity to manufacturing conditions due to its narrow phase diagram, which increases the probability of achieving it with other recurring phases like Bi${}_{25}$FeO${}_{39}$ and Bi${}_{2}$Fe${}_{4}$O${}_{9}$ [5,6,7].

In BFO film production, using an oxygen atmosphere during deposition and further annealing is expected to avoid oxygen vacancies [8] that could generate leakage currents [9]. However, since oxygen is a reactive element, higher partial pressure of this element could increase the presence of spurious unwanted phases [10]. One way to reduce the presence of these phases during the BFO film preparation consists of using a mixed atmosphere of O${}_{2}$ with an inert gas such as argon or nitrogen [11]. One can obtain pure phase BFO thin films over Si (100) by sputtering in an argon atmosphere at low pressure [12]. One way to study the thin film’s surface properties is through X-ray Photoelectron Spectroscopy (XPS). This technique permits the investigation of chemical states based on binding energies (BE) from core level electrons according to atomic bonds in the material.

This work reports on the XPS study of BiFeO${}_{3}$ thin film deposited at a low-pressure argon atmosphere and further etching by Ar${}^{+}$ ions inside the XPS chamber for different times. The etching process allowed us to perform a detailed examination of oxygen vacancies through the core levels analysis of Fe 2p, Bi 4f, O 1s, and the semi-core Bi 5d and not only of O 1s, as conventionally reported. As expected, the XPS analysis presented that Argon ions reduced Fe and Bi, showing additional oxidation states (Fe${}^{2+}$ and Fe${}^{\left(0\right)}$) and (B${}^{\left(0\right)}$), respectively. Although the film deposition in a pure argon atmosphere produces oxygen loss, bringing a nonstoichiometry BFO film, the reduction of valence band maxima (VBM) preserved the BFO semiconductor character, with an absence of Fermi level overlap between the valence and conduction band. This behaviour is interesting for oxygen vacancies controlled photovoltaic effect in some ceramic films.

## 2. Experimental

BiFeO${}_{3}$ thin film was deposited over Si (100) substrate by 35 watts of RF magnetron sputtering, using a commercial BFO target (AJA International, Inc., Scituate, MA, USA). A low-pressure argon atmosphere (work pressure of 3 mTorr) was used to minimize chemical changes and reduce the presence of BFO spurious phases. The substrate deposition temperature was kept at 873 K, with further in-situ annealing at 973 K for 60 min after the film deposition [12]. In grazing incidence mode, the thin-film crystalline structure was studied by high-resolution X-ray diffraction (HR-XRD) performed in the Brazilian Synchrotron Light Laboratory (LNLS), with 1.3776 Å wavelength.

X-ray photoelectron spectroscopy was made using a SPECS PHOIBOS 100/150 spectrometer with a polychromatic Al Kα line (1486.6 eV) with a quartz crystal monochromator. The binding energy scan was at 0.05 eV energy step for high-resolution spectra and 0.5 eV for survey spectra. Quantitative analysis was elaborated using CasaXPS processing software, considering a Shirley background, a Voigt line shape (LA) that better fits each peak, and the instrumental resolution of 0.7 eV measured from FWHM of Au $4f_{7/2}$ peak. The binding energies (BE) were corrected through oxygen core level O1s (529.6 eV) [13,14]; a previous test correction was made using Bi $4f_{7/2}$ (158.7 eV), with no noticeably changes concerning O1s calibration confirming the acceptable oxygen core level correction. For etching depth profiling, argon ions (Ar${}^{+}$) were used at times stages of 40, 80, 200, 600, 2680, and 4480 s. For the sake of clarity, only the 0, 80, and 2680 s spectra will be shown here (Supplementary Material is available with information about the other etching times). Ar${}^{+}$ ions energies were 3 keV from 40 to 200 s, and 5 KeV from 600 to 4480 s. Ar${}^{+}$ etching time at 0 s (spectrum named 0 s or unetched) corresponds to the sample just deposited in a low-pressure argon atmosphere by RF sputtering.

## 3. Results and Discussion

### 3.1. Structural Analysis

**Figure 1:**
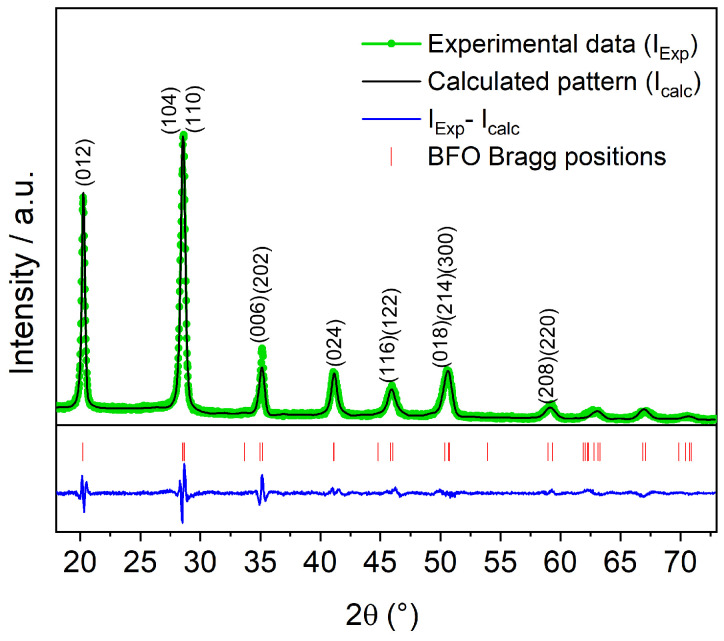
Rietveld refinement plots for the BiFeO${}_{3}$ film deposited in an argon atmosphere at low pressure (unetched sample). The experimental pattern (green dots), the calculated pattern (solid black line), and the difference between them (blue line below patterns) are shown. The calculated Bragg positions are also shown (vertical red lines).

Figure 1 shows the experimental and refined XRD pattern of as-deposited BFO film, named 0 s. The XRD pattern exhibits a polycrystalline film, indexed by ICSD 188396, PDF version 2.4 (2003). The lattice parameters are *a* = *b* = 5.569 Å, and *c* = 13.751 Å, referring to *R3c*-H space group, which represents a rhombohedral structure projected in a hexagonal basis. High-resolution XRD reveals no trace of other phases such Bi${}_{2}$Fe${}_{4}$O${}_{9}$ and Bi${}_{25}$Fe${}_{2}$O${}_{39}$, recurrent in BiFeO${}_{3}$ syntheses [7,15]. The X-ray diffraction refinement, made by the Rietveld method and using Fullprof Suite software [16,17], has shown a selective peak shift for reflections of the type (0k2k), k∈Z. Considering the described selective reflection shifts concerning theoretical Bragg positions, a proper phenomenological model was applied. These shiftings could probably be caused by Si substrate as a result of deposition without a buffer layer, an issue not observed in BFO film deposited over SrTiO${}_{3}$, often used for epitaxial growth.

The film thickness was 90 nm, determined by the X-ray reflectivity technique (XRR); and the calculated crystallite size was around 32 nm, a size below the length of a cycloidal spin structure (62 nm) present in BFO along (110) direction. The interruption of the cycloidal spin structure gives rise to a spin canting that is responsible for a net magnetization [12,18].

### 3.2. Quantitative Atomic Composition

**Figure 2:**
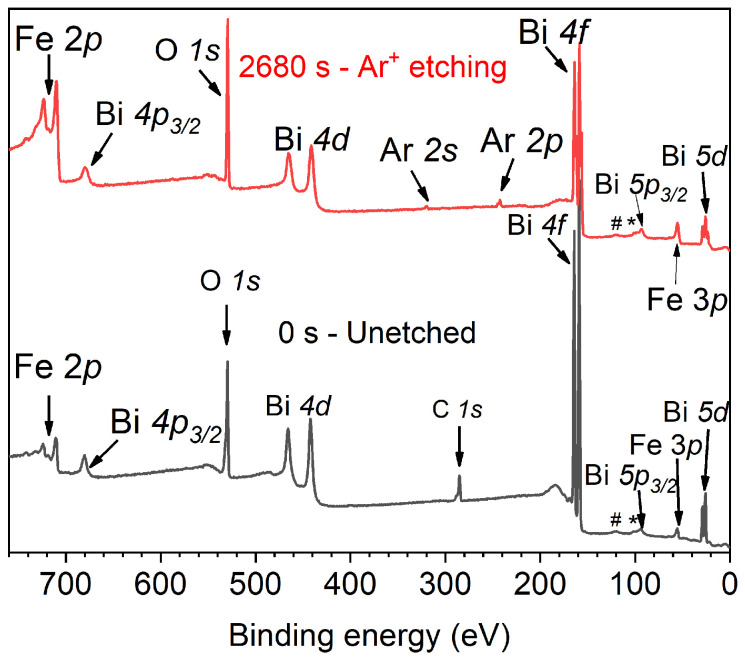
XPS survey spectra of BFO thin-film: (bottom black) unetched and (top red) with 2680 s of argon etching time inside the XPS chamber. The peaks marked with (#) and (*) correspond to Bi $5p_{1/2}$ and Fe 3s respectively.

Figure 2 shows survey spectra at 0 s (unetched) and 2680 s of the Ar${}^{+}$ etching time, with binding energies (BE) ranging from 0 to 760 eV. Both spectra reveal the elements present in BiFeO${}_{3}$, further the adventitious carbon C 1s at 0 s (typical in samples exposed to air). For samples etched at 80 s or more, slight argon implantation is observed, exhibited through Ar 2s (319.9 eV), Ar $2p_{3/2}$ (242.4 eV) and Ar $2p_{1/2}$ (244.6 eV). As expected, those are more visible in the sample etched for 2680 s. Peaks marked with (#) and (*) correspond to Bi $5p_{1/2}$ (120.0 eV) and Fe 3s (99.4 eV), respectively. The highest signal from Bi 4f is due to its large cross-section. Eventually, with the etching time, Bi 4f intensities decrease.

**Figure 3:**
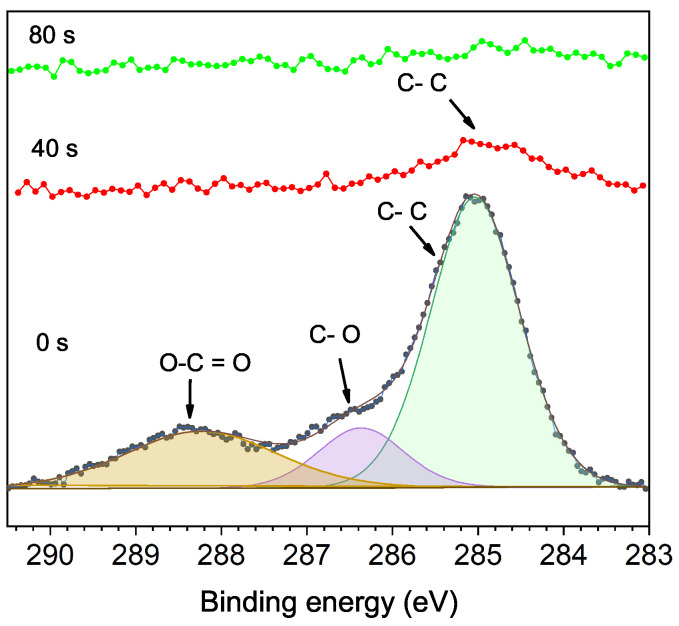
High-resolution XPS spectra showing the evolution of C 1s peaks with Ar${}^{+}$ etching time at 0 s, 40 s and 80 s.

Figure 3 shows the C 1s XPS spectra of the unetched sample and the samples etched at 40 and 80 s. The C 1s peaks correspond to adventitious carbon (AdC), present on all surfaces with air exposure. At 0 s, peaks with a binding energy of 285 eV correspond to C-C bonds, 286.4 eV with C-O bonds, and 288.2 eV with O-C=O bonds. After 40 s, a reduced C-C peak remains, which shifts slightly to 284.7 eV, disappearing after 80 s of etching. The shift of the C-C peak at 40 s and its disappearance after 80 s etching time are why it is not convenient, in this work, to use the C 1s for binding energy correction (charge calibration). Some previous results have already reported issues on the C 1*s* peak for binding energy correction as a not good reference for the calibration of XPS spectra [19,20,21].

**Figure 4:**
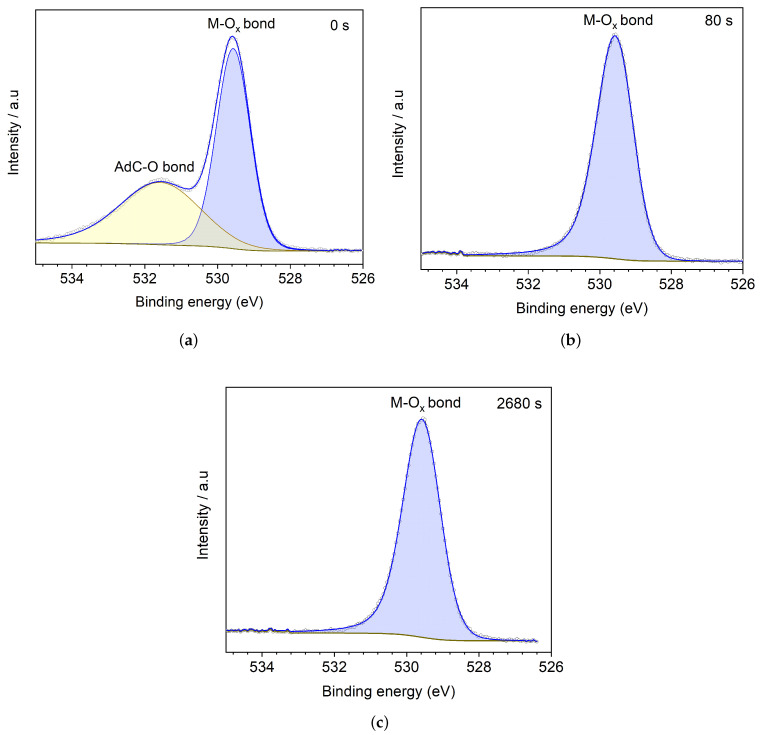
High-resolution XPS spectra from core level O 1s showing the peaks evolution with Ar${}^{+}$ etching time at (**a**) 0 s, (**b**) 80 s and (**c**) 2680 s.

Figure 4 shows high-resolution XPS spectra from core level O 1s, according to etch time at *(a)* 0 s, *(b)* 80 s, and *(c)* 2680 s. Figure 4a shows the XPS spectrum for the unetched BFO film, with a peak at 529.6 eV, corresponding to metal-oxygen bonds (M-O${}_{x}$), and a peak at 531.5 eV, corresponding to oxygen bonded with adventitious carbon (AdC) AdC-O [13], confirmed by the etching process as shown in Figure 4b. The AdC-O peak disappears after 80 s of argon etching, and the metal-oxygen peak preserves line shape, binding energies, and FWHM around 1 eV [22,23,24]. The same behaviour occurs even with the high etching (2680 s), as exhibited in Figure 4c, suggesting that in the pure phase BFO, Bi-O, and Fe-O bonds are related to one photoemission at 529.5 eV.

Some works report oxygen vacancies in XPS through O 1s spectra with a peak around 531.4 eV related to either dangling bonds [25] or photoemissions from Fe-O short bonds in BFO [13] or Bi-O bonds. However, one has to be careful with this assertion. Samples with any exposure to air often present a peak between 531.4–532.0 eV associated with the AdC-O bond. That could also be associated with a shifting influenced by other atoms bonds, for BiFeO${}_{3}$ dopped samples. In this work, the oxygen vacancies in the BFO thin film were studied from bismuth, Fe 2p and valence band spectra. It is recommended to make an argon etching at low energy and with short time exposure in order to ensure a free surface of adventitious carbon to improve the O 1s analysis.

**Figure 5:**
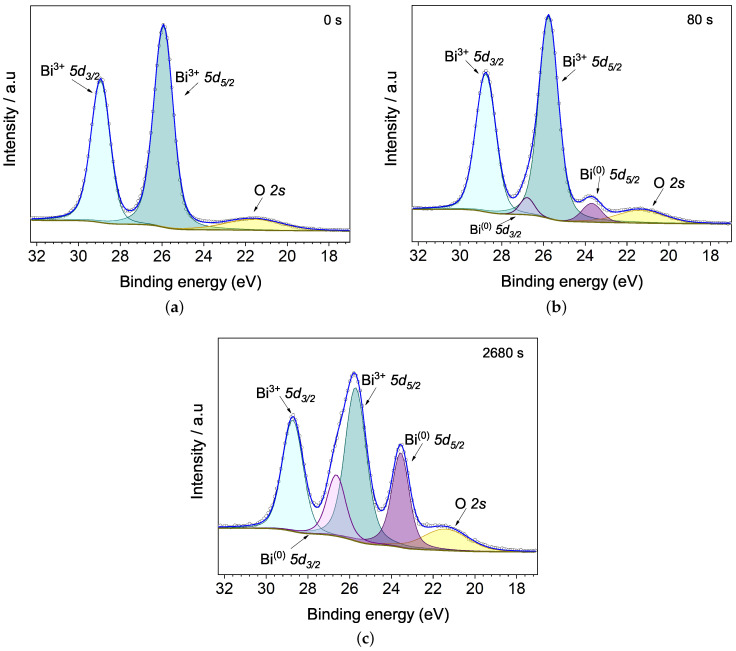
Evolution of Bi 5d and O 2s peaks with Ar${}^{+}$ etching time at (**a**) 0, (**b**) 80 and (**c**) 2680 s. The metallic bismuth doublet peak appears as a function of etching time, indicating a progressive increase in oxygen loss on the BFO surface.

Figure 5 exhibits Bi 5d spectra for the unetched sample and for the 80 and 2680 s Ar${}^{+}$ etched ones. Bi 5d doublet, as consequence of spin orbit splitting, are exhibited being quantified considering peak areas ratio around 2:3, according to degeneracy ratio $2j_{-}+1$:$2j_{+}+1$ [26,27]. At 0 s, the Figure 5a shows the doublet peak with BE around 25.8 eV for Bi${}^{3+}$ $5d_{5/2}$, and 28.8 eV for Bi${}^{3+}$ $5d_{3/2}$, with energetic spin-orbit splitting Δ=3 eV, being these values close to bismuth oxide (Bi${}_{2}$O${}_{3}$) [28,29] and BFO [30].

With etching time, another less intense doublet peak sums to the already identified Bi${}^{3+}$ doublet, the Figure 5b shows Bi${}^{\left(0\right)}\;5d_{5/2}$ and Bi${}^{\left(0\right)}$ $5d_{3/2}$ from metallic bismuth Bi${}^{\left(0\right)}$ with binding energies around 23.7 eV and 26.8 eV, and Δ=3.1 eV [31]. The Bi${}^{\left(0\right)}$ doublet peak concentration values increase considerably with etching time, with 8% of atomic concentration for 80 s and 35% for 2680 s (Figure 5c), indicating an increasing oxygen loss in the BFO surface. According to NIST XPS data base [32], O 2s exhibits a wide range of binding energies between 16 and 32 eV due to O 2s electrons frequently contributing to molecular orbitals [33]. In this work, the O 2s peak from BFO has binding energy at 21.3 eV, a value close to photoelectrons coming from iron oxides such as magnetite and maghemite (21.5 eV) [34].

**Figure 6:**
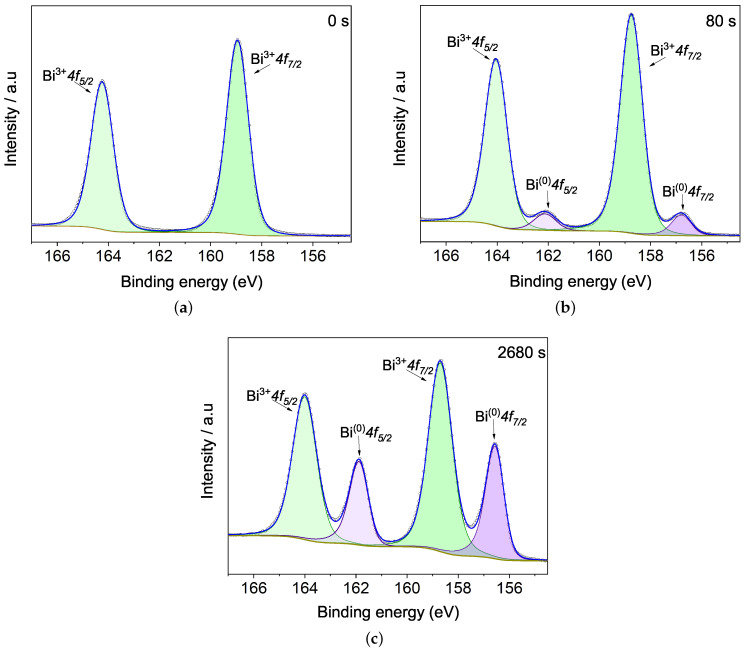
Evolution of Bi 4f doublet peak with Ar${}^{+}$ etching time at (**a**) 0, (**b**) 80 and (**c**) 2680 s. The metallic bismuth doublet peak appears as a function of etching time, indicating a progressive increase in oxygen loss on the BFO surface.

Figure 6 exhibits Bi 4f spectra, being deconvoluted using constrictions in energetic spin-orbit splitting around Δ = 5.3 eV, and areas ratio 3:4 according to degeneracy proportion. Figure 6a exhibits Bi 4f at 0 s, with doublets peak around 158.7 and 164.2 eV corresponding an oxidation state Bi${}^{3+}$. These values agree with other BFO works [13,35], and they are close to bismuth oxide Bi${}_{2}$O${}_{3}$ [36,37]. A peak related to metallic bismuth does not appear (just like in Bi 5d at 0 s) confirming that deposition in a low argon atmosphere is not enough to produce metallic species. Photoemissions coming from Bi 4f at 0 s are higher compared with the other core levels, as the bismuth cross-section is larger than the ones of iron and oxygen and also as 4f energy level takes in more electrons.

Metallic bismuth, Bi${}^{\left(0\right)}$, appears with argon etching time. At 80 s the spectrum shows a doublet metallic bismuth signature (Figure 6b) with BE at 156.8 eV for Bi${}^{\left(0\right)}$ $4f_{7/2}$ and 162.1 eV for Bi${}^{\left(0\right)}$ $4f_{5/2}$, representing 6.2% photoemissions compared with Bi${}^{3+}$. After 2680 s of argon etching (Figure 6c), an enhancement in metallic Bi doublet peak intensity was observed corresponding to 33% of atomic concentration. This value agrees with the one exhibited by Bi 5d spectra, showing the Bi-metallic increase in both core levels in a similar proportion.

Frequently the deconvolution of Fe 2p XPS peaks is mostly complex due to core holes created by photoemissions can produce other couplings in the high-spin states of iron [26], in addition, bonds with other elements, showing frequently asymmetric peaks. For Fe 2p analysis, many works contemplate two ways of XPS deconvolution: multiplet splitting and spin-orbit splitting, the latest a broader method.

Multiple splitting happens when a core electron interacts with an unpaired electron of the upper shell, considering features such as electrostatic interaction, spin-orbit interaction, and crystalline field [26,38,39]; XPS analysis by this procedure is more accurate but is necessary to know how crystalline field influences the transitions between energy states. The argon etching in XPS makes it difficult to know how much the crystalline field was modified in the sample, a reason to consider analysis from spin-orbit splitting in this work purely, stand out that the deconvolution model is based on the deposition in an argon atmosphere, the behaviour of all spectra, and considering satellites parameters related in some works [19,40].

**Figure 7:**
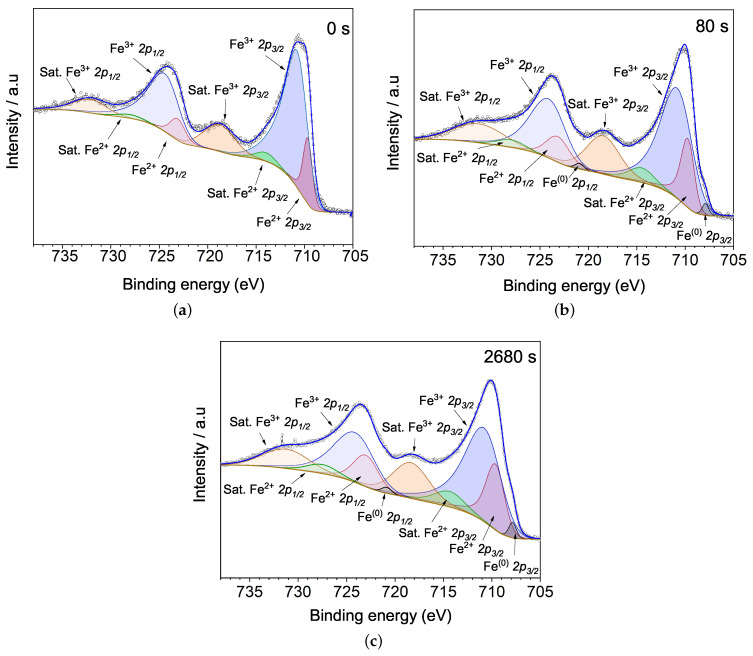
Fe 2p peaks evolution with Ar${}^{+}$ etching time at (**a**) 0 s, (**b**) 80 s and (**c**) 2680 s. The enhancement of the Fe${}^{2+}$ contribution, and the appearance of the metallic iron can be viewed as a function of etching time, indicating a progressive increase in oxygen loss in the BFO surface.

Figure 7 shows the high resolution spectra from Fe 2p core level for the unetched, 80 s and 2680 s etched samples. Doublet from spin-orbit splitting Fe $2p_{1/2}$ and Fe $2p_{3/2}$ were quantified using Δ=13.5 eV, for iron oxidized states [41,42], Δ=13.0 eV, for metallic iron Fe${}^{\left(0\right)}$, and areas ratio 1:2, according to degeneracy proportion [26,27]. There are shake-up satellite peaks for all etching times, representing 2p photoelectron that excites a 3d electron to 4s level [39]. Figure 7a shows the Fe 2p spectrum for the unetched BFO film. The iron has an oxidation state of Fe${}^{3+}$ in BFO, but as a result of oxygen lost during film deposition, Fe${}^{2+}$ emerges, representing with their satellites 17% of Fe 2p photoemissions.

Dual oxidation states of Fe indicate the presence of oxygen vacancies in the film, setting up in the sample Fe${}^{3+}$-O-Fe${}^{2+}$ hybridized states that stabilizes the charge imbalance in the system and magnetic double-exchange interaction appears [12,43]. The binding energies from Fe${}^{3+}$ 2p and Fe${}^{2+}$ 2p are summarized in the Table 1, showing Fe${}^{2+}$ $2p_{3/2}$ and Fe${}^{3+}$ $2p_{3/2}$ peaks with BE of 709.6 and 710.1 eV respectively, close to values reported for BFO [43] and other iron oxides [42,44]. Fe 2p XPS from BFO has a visible shake-up satellites at 718.6 and 731.6 eV corresponding to Fe${}^{3+}$ photoemissions [14], for Fe${}^{2+}$ satellites the binding energies considered was of 713.9 eV and 727.8 eV close to the values reported from magnetite [40,42].

Figure 7b shows Fe 2p XPS spectrum when argon etching time increases to 80 s; the presence of Fe${}^{2+}$ increases to 23%, and metallic iron (Fe${}^{\left(0\right)}$) is exhibited through the doublets with binding energies at 707.9 and 720.9 eV, representing an atomic concentration around 1.0%. After 2680 s of argon etching, Figure 7c, Fe${}^{2+}$ increases to 28% and Fe${}^{\left(0\right)}$ stays almost constant around 1.7%; the oxygen loss is shown here as in Bi 4f and Bi 5d spectra.

**Figure 8:**
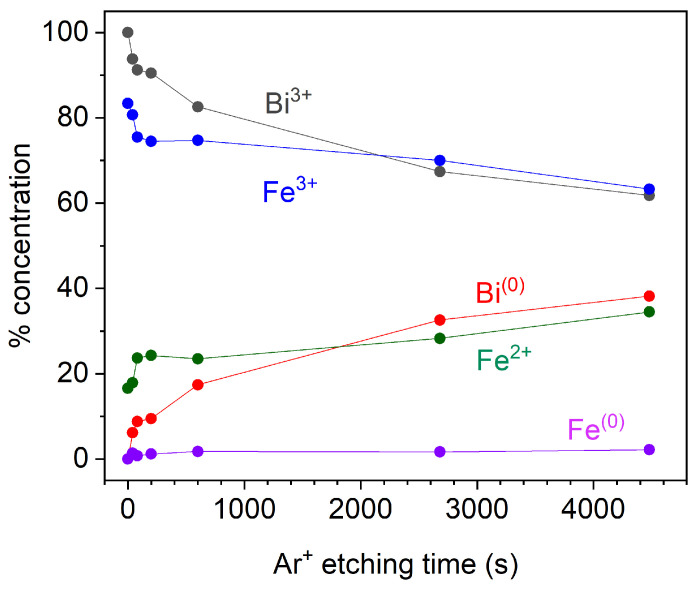
Evolution of Bi 4f and Fe 2p species concentrations with Ar${}^{+}$ etching time.

Atomic concentrations were obtained through CasaXPS software considering relative sensitive factor (RSF) values according to the core level. Figure 8 summarizes the atomic concentrations of species present in Fe 2p and Bi 4f core levels for all etching times. Fe${}^{3+}$ and Bi${}^{3+}$ decrease but still are the main oxidation states in BFO film. Metallic bismuth and Fe${}^{2+}$ concentrations increase with etching time, and at 4480 s they constitute around 44% and 35% of photoemissions respectively. These different values are related to the higher cross-section from bismuth, giving rise to more photoelectrons. Metallic iron and metallic bismuth appear after 40 s of etching time, suggesting that deposition in an argon atmosphere at low pressure could not generate metallic species, but a nonstoichiometric film related to oxygen loss; as consequence, the magnetic behaviour comes from a combination of spin canting by grain size and double exchange interaction [12]. Metallic iron remains constant at around 1.5%, demonstrating that argon etching breaks Fe-O bonds mostly, producing more photoemissions coming from Fe${}^{2+}$ as expected.

The valence band (VB) generally is not quantifiable due to states hybridized present in this region, being a low binding energy zone from the Fermi energy (E${}_{F}$ = BE = 0 eV) to 15 eV [27].

**Figure 9:**
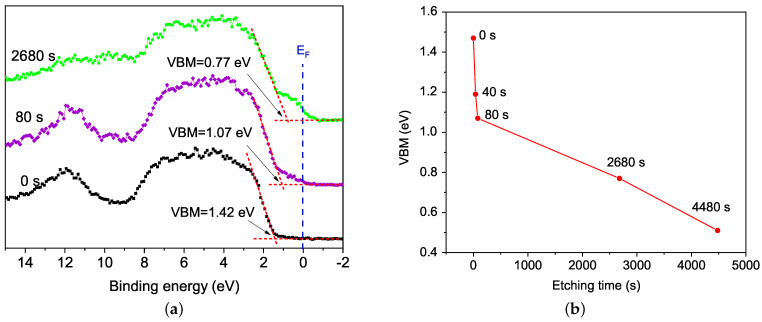
Evolution with Ar${}^{+}$ etching time of: (**a**) Valence band at 0 s, 80 s and 2680 s; (**b**) Valence band maximum (VBM).

Figure 9a exhibits the valence band behaviour after deposition in argon atmosphere (0 s) and with Ar${}^{+}$ etching time inside XPS chamber at 80 s and 2680 s, exhibiting a valence band maximum (VBM). The VBM refers to the valence band edge, being the bandgap in semiconductors defined by the difference between this value and the conduction band minimum (CBM), in this work it is related to the BFO surface.

For the unetched sample, the valence band maximum (VBM) is at 1.42 eV, suggesting maintenance of the semiconductor character in BFO film [5,45], despite the oxygen vacancies generated during BFO deposition in an argon atmosphere. Thus, the BFO film presents a relative band gap reduction compared with VBM typical values between 2.2 eV and 2.6 eV, reported for BFO [46,47,48,49].

While etching time increases, the band gap decreases with a shifting VBM towards the Fermi level. Valence band tails, as a result of conductive states from metallic iron (Fe${}^{\left(0\right)}$) and bismuth (Bi${}^{\left(0\right)}$), appear at 40 s and overstep E${}_{F}$ at 80 s, reaching up to −1 eV value at 2680 s. This behaviour is observed in BFO with α−β and β−γ transitions (rhombohedral to orthorhombic and orthorhombic to cubic phase transitions, respectively), where the semiconducting state goes to a conductive one [5]. Figure 9b shows how the VBM value changes with etching time, being more drastic at the first 80 s and changing more slowly at high etching time, still at 4480 s; the VBM is under the Fermi level. The semiconductor bandgap behaviour with argon etching depends on materials characteristics, like stoichiometry in doped materials [50] or nanostructure character [51,52]. In some semiconductor materials, oxygen vacancies reduce directly the band gap through a shifting of VBM value [50,53,54], aspect seen in this work, suggesting that oxygen vacancies could control the BFO’s semiconductor state, an interesting feature for studies of the photovoltaic effect in BFO.

Binding energies range between 1 and 9 eV, related to the valence band itself, exhibit photo-emissions of hybridized states from Fe 3d, O 2p, and Bi 6p. A peak at 11.8 eV corresponds to lone pair Bi 6s, being more noticeable in the spectra at 0 and 80 s [30]. All the region between 1 and 15 eV has similar behaviour to the density of states (DOS) calculated for BFO [30,55]. Even with high etching time, the main hybridized states are sufficiently preserved.

## 4. Conclusions

We report the XPS study of argon sputtering on pure phase BFO film surface through the Bi 4f and 5d, O 1s, Fe 2p components, and its valence band. The effects of a low-pressure argon atmosphere on BFO deposition and argon etching inside the XPS chamber at different times were also investigated. Contrary to the usual reported BFO deposition methods, using an oxygen-rich atmosphere, the low-pressure argon atmosphere is proved to be efficient to produce pure phase BFO, without other phases like Bi${}_{2}$Fe${}_{4}$O${}_{9}$ and Bi${}_{25}$FeO${}_{39}$, and oxygen loss brings a nonstoichiometric film. Oxygen vacancies are revealed through the presence of Fe${}^{2+}$ and a reduced valence band maximum, without traces of metallic species that could lead to misinterpretations about ferromagnetic behaviour in BFO films. Subsequent Ar${}^{+}$ etching inside the XPS chamber increases Fe${}^{2+}$ and promotes the appearance of metallic Fe and metallic Bi, as expected. The band gap reduction through the narrowing of valence band maxima shows that the pure BFO phase obtained still preserves the semiconductor character. This behaviour is interesting for oxygen vacancies controlled photovoltaic effect in some ceramic films.

## Figures and Tables

**Table 1:** Binding energies from Fe 2p core level.

Fe 2p Peak	Binding Energy (eV)
Fe${}^{2+}$ $2p_{3/2}$	709.6
Fe${}^{3+}$ $2p_{3/2}$	710.1
Fe${}^{2+}$sat. $2p_{3/2}$	713.9
Fe${}^{3+}$sat. $2p_{3/2}$	718.6
Fe${}^{2+}$ $2p_{1/2}$	723.1
Fe${}^{3+}$ $2p_{1/2}$	723.6
Fe${}^{2+}$sat. $2p_{1/2}$	727.8
Fe${}^{3+}$sat. $2p_{1/2}$	731.6
Fe${}^{\left(0\right)}$ $2p_{3/2}$	707.9
Fe${}^{\left(0\right)}$ $2p_{1/2}$	720.9

## Data Availability

Not applicable.

## Supplementary Materials

The following are available at https://www.mdpi.com/article/10.3390/ma15124285/s1. It displays the XPS spectra and peak parameter evolution with Ar${}^{+}$ etching time from core levels C 1s, O 1s, Bi 5d, Bi 4f, Fe 2p and the valence band.

## Abbreviations

The following abbreviations are used in this manuscript:

BFO	Bismuth ferrite- BiFeO${}_{3}$
VB	Valence band
VBM	Valence band maximum
