# Supplementary material for: XPS Study in BiFeO3 Surface Modified by Argon Etching
Source: Materials (Basel). 2022 Jun 17;15(12):4285. doi: 10.3390/ma15124285 (PMC9227888; doi:10.3390/ma15124285)
Supplement: Supplementary file 1 [file materials-15-04285-s001.zip › materials-1695419-supplementar materials.pdf]

Article

XPS study in $BiFeO_3$ surface modified by argon etching

Supplementary information

G.A. Gómez Iriarte [1], A. Pentón-Madrigal [2] L.A.S. de Oliveira [3] and J.P. Sinnecker [1]

[1] Centro Brasileiro de Pesquisas Físicas, Rua Xavier Sigaud 150, Rio de Janeiro 22290-180, RJ, Brazil; grecia@cbpf.br; sinnecker@cbpf.br

[2] Facultad de Física, Universidad de La Habana, La Habana CP 10400, Cuba; arbelio@fisica.uh.cu

[3] Núcleo Multidisciplinar de Pesquisas em Nanotecnologia, Universidade Federal do Rio de Janeiro, Rodovia Washington Luiz, n. 19593, km 104,5, Duque de Caxias 25240-005, RJ, Brazil; laso@ufrj.br

1. Adventitious carbon C *1s* (AdC)

Figure S1 shows the behavior of C 1*s* peaks of adventitious carbon with argon etching until 80 s when the film is carbon-free. The table S1 shows the evolution of the peak parameters over-etching time, the data at 80 s are missing because the statistic is too low for quantification.

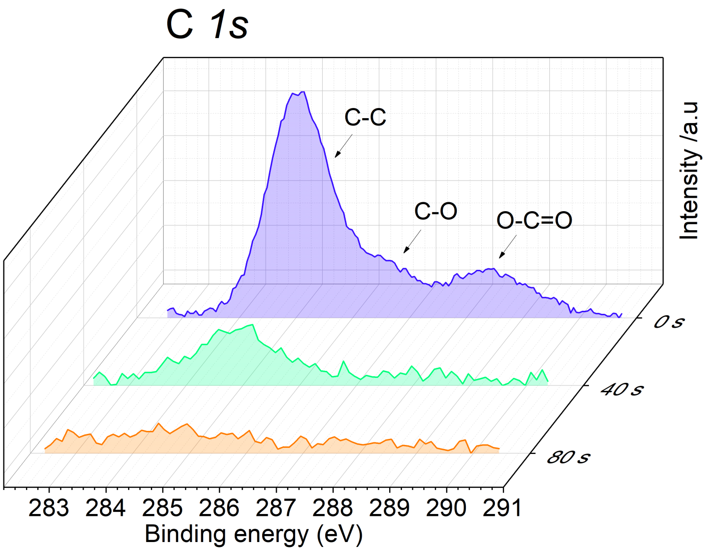

Figure S1. C 1*s* XPS spectra evolution with Ar^+ etching time at 0 s, 40 s and 80 seconds.

Table S1. C 1*s* peaks parameters evolution with Ar^+ etching time. R.S.F= 1.0

Etching time (s)	C 1*s* peak	Binding energy (eV)	FWHM (eV)	Area (a.u^2)	Area (%)
0	C-C	285.0	1.2	11836.2	65.1
	C-O	286.4	1.2	2342.5	12.9
	O-C=O	288.2	2.1	4003.1	22.0
40	C-C	284.7	1.6	2517.7	65.0
	C-O	286.0	1.7	755.3	19.5
	O-C=O	287.9	1.8	602.9	15.5

2. O 1*s* core level

Figure S2 shows the evolution of O 1*s* peaks with argon etching. The table S2 exhibits the development of peak parameters with etching time. The adventitious carbon disappears at 80 s already.

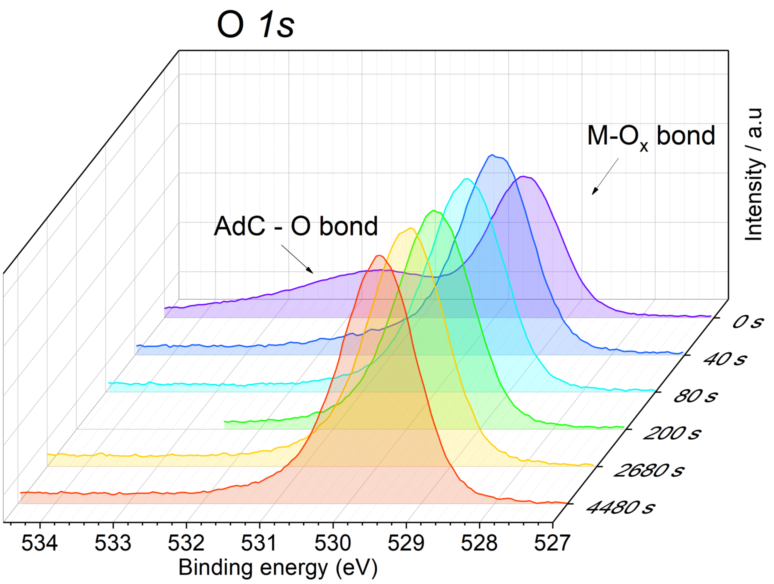

Figure S2. O 1*s* XPS spectra evolution with Ar^+ etching time.

Table S2. O 1*s* peaks parameters evolution with Ar^+ etching time. R.S.F= 2.93

Etching time (s)	O 1*s* peak	Binding energy (eV)	FWHM (eV)	Area (a.u^2)	Area (%)
0	M-O$_x$<br>AdC-O	529.6<br>531.5	1.1<br>2.6	49486.4<br>38096.7	56.5<br>43.5
40	M-O$_x$<br>AdC-O	529.6<br>530.9	1.2<br>1.6	54689.4<br>7609.3	87.8<br>12.2
80	M-O$_x$	529.5	1.2	89565.3	100
200	M-O$_x$	529.6	1.2	87284.1	100
2680	M-O$_x$	529.5	1.2	81339.5	100
4480	M-O$_x$	529.5	1.2	79750.0	100

3. Bi 5*d* core level

Figure S3 shows the behaviour of Bi 5*d* peaks with argon etching and Table S3 summa-rizes the evolution of the peak parameters. The oxidation state of Bi^{3+} decreases visibly with argon etching time, unlike the bismuth metal $Bi^{(0)}$, which appears at 40 s.

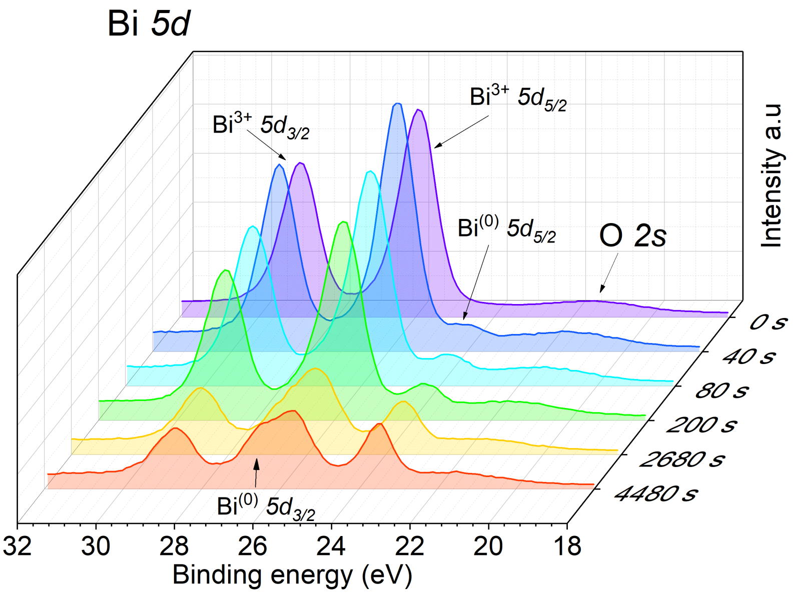

Figure S3. Bi 5*d* XPS spectra evolution with Ar^+ etching time.

Table S3. Bi 5*d* peaks parameters evolution with Ar^+ etching time. R.S.F = 3.0

Etching time (s)	Bi 5*d* peak	Binding energy (eV)	FWHM (eV)	Area (a.u^2)	Area (%)
0	Bi^{3+} $5d_{5/2}$	25.8	1.1	35580.5	59.9
	Bi^{3+} $5d_{3/2}$	28.8	1.1	23838.9	40.1
40	Bi^{3+} $5d_{5/2}$	25.8	1.1	28964.2	57.1
	Bi^{3+} $5d_{3/2}$	28.8	1.1	19406.0	38.2
	$Bi^{(0)}$ $5d_{5/2}$	24.0	1.0	1478.6	2.9
	$Bi^{(0)}$ $5d_{3/2}$	27.1	0.9	916.8	1.8
80	Bi^{3+} $5d_{5/2}$	25.8	1.1	38155.1	55.5
	Bi^{3+} $5d_{3/2}$	28.8	1.1	25182.4	36.6
	$Bi^{(0)}$ $5d_{5/2}$	23.7	1.1	3271.2	4.8
	$Bi^{(0)}$ $5d_{3/2}$	26.8	0.8	2178.6	3.1
200	Bi^{3+} $5d_{5/2}$	25.8	1.1	35562.6	54.0
	Bi^{3+} $5d_{3/2}$	28.8	1.1	23826.9	36.2
	$Bi^{(0)}$ $5d_{5/2}$	23.7	1.1	3858.4	5.9
	$Bi^{(0)}$ $5d_{3/2}$	26.8	0.8	2569.7	3.9

Etching time (s)	Bi $5d$ peak	Binding energy (eV)	FWHM (eV)	Area (a.u^2)	Area (%)
2680	Bi^{3+} $5d_{5/2}$	25.7	1.2	15235.0	38.8
	Bi^{3+} $5d_{3/2}$	28.7	1.2	10207.5	26.0
	$Bi^{(0)}$ $5d_{5/2}$	23.7	1.0	8271.4	21.1
	$Bi^{(0)}$ $5d_{3/2}$	26.6	1.1	5508.7	14.1
4480	Bi^{3+} $5d_{5/2}$	25.7	1.2	12576.8	33.4
	Bi^{3+} $5d_{3/2}$	28.7	1.2	8426.5	22.4
	$Bi^{(0)}$ $5d_{5/2}$	23.5	0.9	10029.5	26.6
	$Bi^{(0)}$ $5d_{3/2}$	26.6	0.8	6679.7	17.6

4. Bi $4f$ core level

Figure S4 shows the behavior of Bi $4f$ peaks with argon etching and the table S4 sum-marizes the evolution of peak parameters. Bi^{3+} oxidation state is visible decreasing with argon etching time as opposed to bismuth metallic, $Bi^{(0)}$, that appears at 40 s.

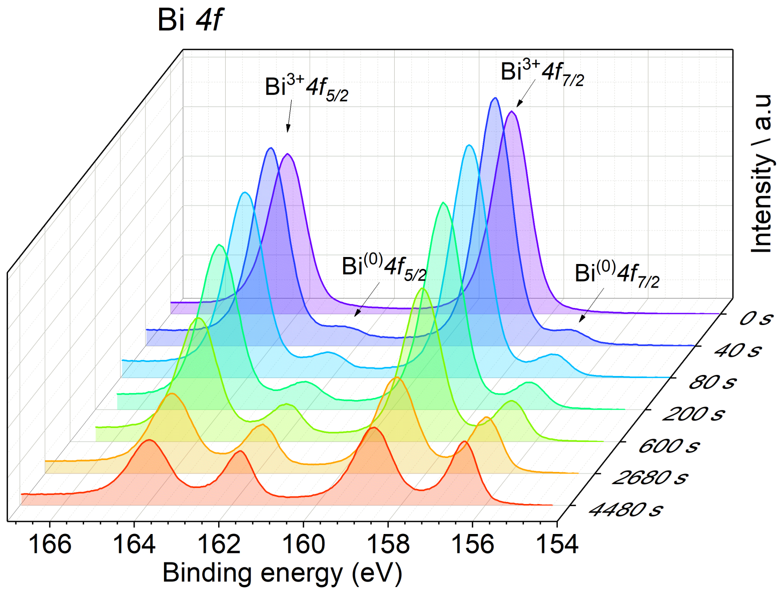

Figure S4. Evolution of Bi $4f$ doublet peak with Ar^+ etching time. The metallic bismuth doublet peak appears as a function of etching time, indicating a progressive increase in oxygen loss in the BFO surface.

Table S4. Bi 4f peaks parameters evolution with Ar^+ etching time. R.S.F = 24.8

Etching time (s)	Bi 4f peak	Binding energy (eV)	FWHM (eV)	Area (a.u^2)	Area relative(%)
0	Bi^{3+} 4$f_{7/2}$	158.7	1.0	184552.6	56.6
	Bi^{3+} 4$f_{5/2}$	164.2	1.0	141551.7	43.4
40	Bi^{3+} 4$f_{7/2}$	158.7	1.0	153674.5	53.6
	Bi^{3+} 4$f_{5/2}$	164.0	1.0	115255.8	40.2
	$Bi^{(0)}$ 4$f_{7/2}$	157.0	1.1	9971.6	3.5
	$Bi^{(0)}$ 4$f_{5/2}$	162.3	1.1	7777.8	2.7
80	Bi^{3+} 4$f_{7/2}$	158.7	1.0	212134.4	52.1
	Bi^{3+} 4$f_{5/2}$	164.0	1.0	159100.8	39.1
	$Bi^{(0)}$ 4$f_{7/2}$	156.8	0.9	20059.2	4.9
	$Bi^{(0)}$ 4$f_{5/2}$	162.1	1.0	15646.2	3.9
200	Bi^{3+} 4$f_{7/2}$	158.8	1.0	194516.5	51.7
	Bi^{3+} 4$f_{5/2}$	164.1	1.0	145887.4	38.8
	$Bi^{(0)}$ 4$f_{7/2}$	156.7	0.9	20251.7	5.4
	$Bi^{(0)}$ 4$f_{5/2}$	162.0	0.9	15796.3	4.1
600	Bi^{3+} 4$f_{7/2}$	158.7	1.1	151455.5	47.2
	Bi^{3+} 4$f_{5/2}$	164.0	1.1	113591.6	35.4
	$Bi^{(0)}$ 4$f_{7/2}$	156.6	0.8	31331.1	9.8
	$Bi^{(0)}$ 4$f_{5/2}$	161.7	0.9	24438.3	7.6
2680	Bi^{3+} 4$f_{7/2}$	158.7	1.1	81766.5	38.5
	Bi^{3+} 4$f_{5/2}$	164.0	1.1	61324.8	28.9
	$Bi^{(0)}$ 4$f_{7/2}$	156.5	0.8	39337.0	18.5
	$Bi^{(0)}$ 4$f_{5/2}$	161.8	0.9	29896.2	14.1
4480	Bi^{3+} 4$f_{7/2}$	158.6	1.1	67157.1	35.2
	Bi^{3+} 4$f_{5/2}$	163.9	1.1	50703.6	26.6
	$Bi^{(0)}$ 4$f_{7/2}$	156.5	0.8	41866.2	21.9
	$Bi^{(0)}$ 4$f_{5/2}$	161.8	0.8	31232.2	16.3

5. Fe 2*p* core level

Figure S5 shows the behavior of Fe 2*p* peaks with argon etching and the table S5 summa-rizes the evolution of peak parameters. A deconvolution model describes the presence of Fe^{2+} in the film and the appearance of metallic $Fe^{(0)}$ certainly at 40 s.

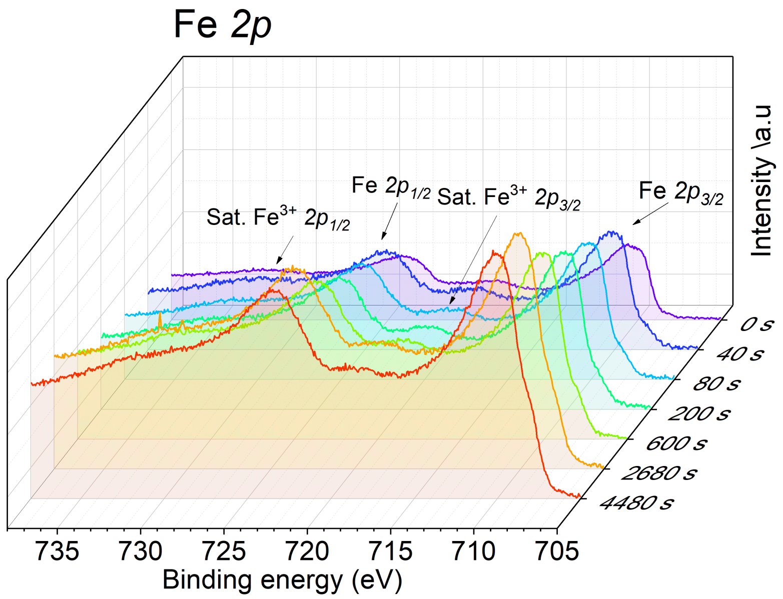

Figure S5. Fe 2*p* evolution with Ar^+ etching time.

Table S5. Fe 2*p* peaks parameters evolution with Ar^+ etching time. R.S.F= 16.4

Etching time (s)	Fe 2*p* peak	Binding energy (eV)	FWHM (eV)	Area (a.u^2)	Area (%)
0	Fe^{2+} $2p_{3/2}$	709.6	1.1	8470.3	8.4
	Fe^{2+} $2p_{1/2}$	723.1	1.8	4658.7	4.5
	$Fe^{2+}_{Sat.}$ $2p_{3/2}$	713.9	2.5	2541.1	2.5
	$Fe^{2+}_{Sat.}$ $2p_{1/2}$	727.8	2.9	1164.7	1.2
	$Fe^{3+}_{Sat.}$ $2p_{3/2}$	718.6	3.7	10896.2	10.7
	$Fe^{3+}_{Sat.}$ $2p_{1/2}$	731.6	3.9	5992.9	6.0
	Fe^{3+} $2p_{3/2}$	710.1	2.5	43584.7	43.0
	Fe^{3+} $2p_{1/2}$	723.6	3.3	23971.6	23.7

Etching time (s)	Fe $2p$ peak	Binding energy (eV)	FWHM (eV)	Area (a.u^2)	Area (%)
40	$Fe^{(0)}\ 2p_{3/2}$	707.9	1.1	1333.8	0.9
	$Fe^{(0)}\ 2p_{1/2}$	720.9	1.2	746.9	0.5
	$Fe^{2+}\ 2p_{3/2}$	709.6	1.4	12815.2	8.4
	$Fe^{2+}\ 2p_{1/2}$	723.1	2.2	7176.5	4.7
	$Fe^{2+}_{Sat.}\ 2p_{3/2}$	714.1	2.7	4869.8	3.2
	$Fe^{2+}_{Sat.}\ 2p_{1/2}$	727.8	3.0	2434.9	1.6
	$Fe^{3+}\ 2p_{3/2}$	709.9	3.3	56392.8	36.8
	$Fe^{3+}\ 2p_{1/2}$	723.4	3.8	31579.9	20.7
	$Fe^{3+}_{Sat.}\ 2p_{3/2}$	718.4	4.7	21429.3	14.0
	$Fe^{3+}_{Sat.}\ 2p_{1/2}$	730.9	5.9	14098.2	9.2
80	$Fe^{(0)}\ 2p_{3/2}$	707.9	0.9	1402.9	0.5
	$Fe^{(0)}\ 2p_{1/2}$	720.9	1.2	785.9	0.3
	$Fe^{2+}\ 2p_{3/2}$	709.6	1.6	28696.5	11.0
	$Fe^{2+}\ 2p_{1/2}$	723.1	2.8	16357.0	6.3
	$Fe^{2+}_{Sat.}\ 2p_{3/2}$	714.4	3.7	10904.7	4.2
	$Fe^{2+}_{Sat.}\ 2p_{1/2}$	727.8	3.9	5739.3	2.2
	$Fe^{3+}\ 2p_{3/2}$	710.0	3.6	91355.5	34.9
	$Fe^{3+}\ 2p_{1/2}$	724.0	3.9	51159.1	19.6
	$Fe^{3+}_{Sat.}\ 2p_{3/2}$	718.4	4.2	34715.1	13.3
	$Fe^{3+}_{Sat.}\ 2p_{1/2}$	730.8	5.4	20098.2	7.7
200	$Fe^{(0)}\ 2p_{3/2}$	707.9	0.9	2205.3	0.8
	$Fe^{(0)}\ 2p_{1/2}$	720.9	1.2	1234.9	0.4
	$Fe^{2+}\ 2p_{3/2}$	709.6	1.7	33483.3	11.4
	$Fe^{2+}\ 2p_{1/2}$	723.1	2.8	18750.6	6.4
	$Fe^{2+}_{Sat.}\ 2p_{3/2}$	714.4	3.4	12723.6	4.3
	$Fe^{2+}_{Sat.}\ 2p_{1/2}$	727.6	3.6	6361.8	2.2
	$Fe^{3+}\ 2p_{3/2}$	710.0	3.6	100283.6	34.0
	$Fe^{3+}\ 2p_{1/2}$	723.5	3.9	56158.8	19.1
	$Fe^{3+}_{Sat.}\ 2p_{3/2}$	718.4	4.2	38107.7	12.9
	$Fe^{3+}_{Sat.}\ 2p_{1/2}$	731.0	6.0	25070.9	8.5
600	$Fe^{(0)}\ 2p_{3/2}$	707.8	1.1	4314.2	1.2
	$Fe^{(0)}\ 2p_{1/2}$	720.9	1.2	2416.0	0.6
	$Fe^{2+}\ 2p_{3/2}$	709.5	1.8	40189.5	11.0
	$Fe^{2+}\ 2p_{1/2}$	722.9	2.9	22506.1	6.2
	$Fe^{2+}_{Sat.}\ 2p_{3/2}$	714.3	4.6	15272.0	4.2
	$Fe^{2+}_{Sat.}\ 2p_{1/2}$	727.5	3.4	7636.0	2.1
	$Fe^{3+}\ 2p_{3/2}$	709.8	3.4	124271.7	34.1
	$Fe^{3+}\ 2p_{1/2}$	723.3	4.2	69592.1	19.2
	$Fe^{3+}_{Sat.}\ 2p_{3/2}$	718.2	4.1	47223.2	13.0
	$Fe^{3+}_{Sat.}\ 2p_{1/2}$	730.9	6.0	29825.2	8.4

Etching time (s)	Fe $2p$ peak	Binding energy (eV)	FWHM (eV)	Area (a.u^2)	Area (%)
2680	$Fe^{(0)}$ $2p_{3/2}$	707.9	1.0	4867.4	1.1
	$Fe^{(0)}$ $2p_{1/2}$	720.9	1.2	2677.1	0.6
	Fe^{2+} $2p_{3/2}$	709.6	2.2	59954.1	13.5
	Fe^{2+} $2p_{1/2}$	723.0	2.9	33574.3	7.6
	$Fe^{2+}_{Sat.}$ $2p_{3/2}$	714.4	3.7	21583.5	4.9
	$Fe^{2+}_{Sat.}$ $2p_{1/2}$	727.6	4.5	11391.3	2.3
	Fe^{3+} $2p_{3/2}$	709.8	4.2	142689.6	32.1
	Fe^{3+} $2p_{1/2}$	723.3	4.6	79906.2	18.1
	$Fe^{3+}_{Sat.}$ $2p_{3/2}$	718.3	4.6	52795.2	11.9
	$Fe^{3+}_{Sat.}$ $2p_{1/2}$	731.1	5.5	34245.5	7.9
4480	$Fe^{(0)}$ $2p_{3/2}$	707.9	1.2	5795.0	1.5
	$Fe^{(0)}$ $2p_{1/2}$	720.9	1.3	2897.5	0.7
	Fe^{2+} $2p_{3/2}$	709.5	2.4	64262.8	16.3
	Fe^{2+} $2p_{1/2}$	722.9	3.0	35987.2	9.2
	$Fe^{2+}_{Sat.}$ $2p_{3/2}$	714.3	4.0	116610.2	5.9
	$Fe^{2+}_{Sat.}$ $2p_{1/2}$	727.5	4.4	12209.9	3.1
	Fe^{3+} $2p_{3/2}$	709.9	4.1	116610.2	29.6
	Fe^{3+} $2p_{1/2}$	723.4	4.9	65301.7	16.7
	$Fe^{3+}_{Sat.}$ $2p_{3/2}$	718.4	4.4	43145.8	11.1
	$Fe^{3+}_{Sat.}$ $2p_{1/2}$	731.2	4.4	23322.0	5.9

6. Valence Band

Figure S6 and table S6 show the evolution of $BiFeO_3$ valence band region with argon etching time. Bi 6s peak is noticeable until an etching time of 80 s; over 2680 s is possible that Bi 6s are in new states hybridized with the other elements present in the film. The valence band region, between 2 eV and 8 eV, keeps stable with a reduction of valence band maximum (VBM), also the increase of valence tails representing metallic states from iron and bismuth.

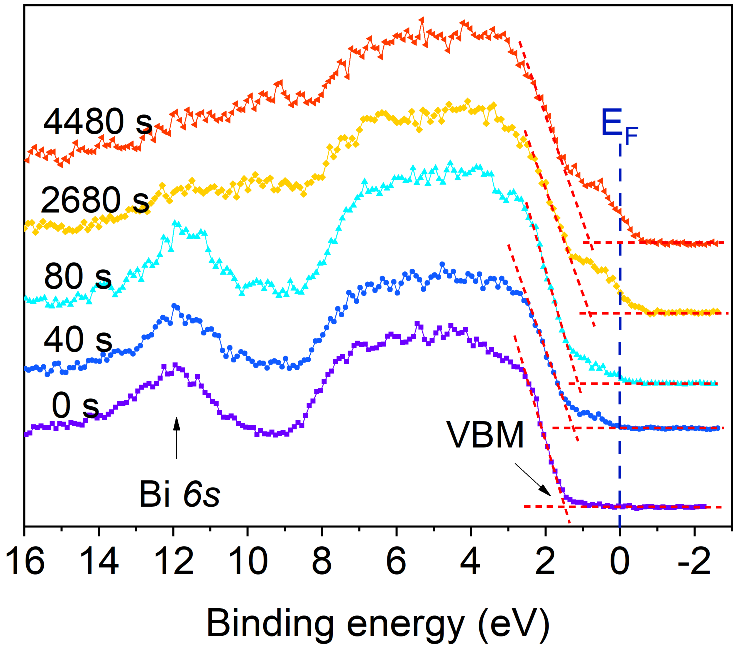

Figure S6. $BiFeO_3$ valence band behavior with Ar^+ etching time

Table S6. $BiFeO_3$ valence band maximum evolution with Ar^+ etching time.

Etching time (s)	Binding energy (eV) Bi 6s	Valence Band Maximum (eV) (VBM)
0	11.8	1.47
40	11.8	1.19
80	11.6	1.07
2680	-	0.77
4480s	-	0.51
